# Start with the truth: lessons from the decolonizing birth research project

**DOI:** 10.3389/fgwh.2026.1780730

**Published:** 2026-10-30

**Authors:** Rachel Olson, Wanda Phillips-Beck, Erin Tomkins, Elder Roberta Price, Saraswathi Vedam

**Affiliations:** 1 Tr'ondëk Hwëch'in First Nation and Firelight, West Vancouver, BC, Canada; 2 Indigenous Research Chair in Nursing, University of Manitoba, First Nations Health and Social Secretariat of Manitoba, Winnipeg, MB, Canada; 3 Firelight, Vancouver, BC, Canada; 4 Snuneymuxw and Cowichan Nations, Richmond, BC, Canada; 5 Birth Place Lab, Midwifery, Faculty of Medicine, University of British Columbia, Vancouver, BC, Canada

**Keywords:** anti-Indigenous racism, childbirth, colonial violence, decolonizing research, epistemic justice, health equity, Indigenous population, Indigenous research

## Abstract

Indigenous families in Canada have long endured colonial violence in birthing spaces, including systemic racism, forced evacuations, and child welfare interventions. Parallel to these harms, academic research has often functioned as a tool of colonialism. The Decolonizing Birth Research (DBR) project, conducted between 2019 and 2022 across six Indigenous communities in British Columbia and Manitoba, sought to challenge these legacies by centering Indigenous leadership, knowledge systems, and governance through a community-based, participatory model. Two Indigenous scholars, with lived experience, anthropologic, and clinical research expertise in perinatal health services, as well as community-specific knowledge, were supported by an Elder and an academic midwife to co-lead this project. Having an Indigenous research organization as an integral part of the research team allowed the project to navigate and, in some cases, circumvent the colonial constraints of academic institutions, highlighting the capacity of Indigenous organizations to support communities in conducting rigorous, ethical, and culturally grounded research. This article explores both the innovations and structural barriers encountered throughout the project, offering a critical reflection on how academia continues to perpetuate epistemic and structural violence despite commitments to reconciliation. It calls for a reimagining of perinatal and health equity research as a practice that can and must be led by Indigenous communities, both within and beyond the academy.

## Introduction

### Background

Understanding colonial violence in Indigenous families’ experiences of birth is a complicated endeavour, and at the same time, a very straightforward exercise. This paper aims to address not only how community-led birth research contributes to the understanding of how birth and perinatal services are experienced by Indigenous families in Canada, but also critically examine the research process itself with a decolonial lens, and show how these forms of colonial violence constantly challenge both experiences of birth and the undertaking of birth research. For many generations, Indigenous families have faced colonial violence within birthing systems and spaces, whether it be forced birth evacuations, racism from providers, or interventions from the child welfare system. The distress and racism experienced by Indigenous people during birth, including discrimination, loss of autonomy and dehumanizing interactions with care providers has been documented (1–3). Pregnant Indigenous women, families and Knowledge Keepers report a lack of culturally safe care, systemic discrimination, coercion, and dismissal of concerns that have led to a mistrust in the healthcare system, and urgent calls for integration of Indigenous knowledge and traditions in perinatal services (4).

Recent research confirms that Indigenous, Black, and People of Color experienced more adverse health outcomes (5–7), and significantly more disrespect and coercion when engaging with perinatal services in North America (2, 5, 8). Findings from the national RESPCCT Study confirm that Indigenous individuals in Canada disproportionately experience a loss of autonomy, disrespect and mistreatment during pregnancy and childbirth (3). Experiences of trauma during pregnancy and birth, discrimination, and racism among marginalized groups such as Indigenous women, have been linked to adverse mental health and perinatal health outcomes for both mothers and infants (9–11). Investigators find that structural inequities reduce uptake of perinatal and mental health services (12–14). However, until recently research on the drivers of these disparities in perinatal outcomes and experience has been scarce. Moreover, large population-based studies fail to use decolonized, participatory approaches nor person-centred measures that have been validated by Indigenous service users. The national RESPCCT study aimed to address some of these methodological challenges.

Western research has long been a key feature of and contributor to colonial oppression. Historically, research has not benefited Indigenous people, has been unethical, and inflicted harm on Indigenous people (15). As a result, research projects are frequently viewed with “suspicion and hostility as something intrusive, exploitative, and unethical” (16). The concept of decolonizing research methodologies is not new. Foundational work by Indigenous scholars have critiqued existing modes of research in Indigenous communities and signal a move towards research that centers Indigenous methodologies (17–21). At the same time, the critique of co-opting the language of decolonization without addressing the very structures from within which academic research operates is an important one. This paper attempts to ground what we mean by decolonizing research in concrete and substantive ways, not just as a “metaphor” (20). We use the concept of decolonizing research as a vehicle to explore the intentional redistribution of power, decision-making, and resources to Indigenous communities in the project, as well as governance processes that uphold Indigenous self-determination over research methods, community engagement, and data sovereignty. In this paper, we describe how the commitment to realizing decolonial research shaped not only how the research was conducted and led in the communities, but also how broader institutional constraints were navigated and overcome.

For research to be culturally safe, it must be based on respect for the individuals who participate in a research project (22, 23). As Wilson & Neville (2009) explain; “culturally safe research practice is primarily determined by those groups who are researched—not by the researchers themselves. When applied to research, cultural safety begins from the inception of a research idea and extends to the dissemination of the findings. It is about research participants feeling included, respected, and that they can trust the researchers and what they will do with the information shared with them.”

It is critically important to understand experiences of racism described by Indigenous peoples as reflecting overlaying racist discourses, policies and practices, which are firmly entrenched in organizations and institutions, and in the dominant society through the media, public conversations, and everyday practices (24). This understanding is the first step towards accountability, redress, and improved quality of care. Recognizing this deeply rooted legacy of harm, both in birthing practices and in the conduct of research, it becomes essential to reimagine how knowledge is generated in ways that uphold Indigenous sovereignty and well-being (25, 26).

Within this context, the research team undertook a community-based, participatory research model across six Indigenous communities—five in BC and one in Manitoba—that foregrounded Indigenous leadership, knowledge systems, and self-determination in the investigation of respectful maternity care (27). The project was entitled, “Decolonizing Birth Research: Indigenous researchers, clinicians, and communities measuring respect during childbearing” (DBR). The project’s primary objectives were to identify community-defined indicators of respect, disrespect, racism, and mistreatment during pregnancy and childbirth, and to demonstrate decolonizing methodologies in patient-oriented research. In addition to these aims, the reality of attempting to undertake a decolonized approach to research within the institutional constraints posed during the research process soon became a main focus of the lead researchers and led the project team to continually have to find innovative ways to stay committed to the values set out at the beginning of the project. Therefore, in the paper, we are focusing on the methodological and structural aspects of the process, rather than the primary research outcomes. The paper focuses on interviews with those involved in conducting the project, and reflections on the overall process. In this way, we hope to contribute to the articulation of the ongoing need for decolonized perinatal research processes, lessons learned, and new insights on the way forward to justice-based research in service of Indigenous families.

## Governance, procedures, settings

Undertaken from the fall of 2019 to spring of 2022, the Decolonizing Birth Research (DBR) project sought to confront these legacies of harm by centering Indigenous leadership, knowledge systems, and self-determination in both the content and process of research. The project was led by Indigenous researchers, Dr. Rachel Olson (Tr’ondek Hwech’in First Nation), President and Director of Firelight and Dr. Wanda Phillips-Beck (Hollow Water First Nation), Seven Generations Scholar, First Nations Health and Social Secretariat of Manitoba (FNHSSM), with the support of Elder Roberta Price (Snuneymuxw and Cowichan Nations) and Dr. Saraswathi Vedam, Principal of the Birth Place Lab (BPL) at the University of British Columbia (UBC). The research was funded through the Support for Patient Oriented Research (SPOR) Support Unit in British Columbia (BC). Ethics approval was provided by the University of British Columbia Behavioural Ethics Board (#H2001070).

### Indigenous governance

We convened a multi-stakeholder Steering Council (SC) with ceremony and the leadership of an Elder at the opening of the project. The Co-PIs and SC agreed upon study design, and identified five-six potential communities to engage. The Co-PIs and Elder and continued to meet monthly over the next two years to provide strategic oversight and direction, troubleshoot obstacles, and allocate portfolios of work to Firelight or the Birth Place Lab always considering issues of capacity, trust, relationships, and community responsiveness.

They enlisted the support of the BPL Operations and Equity staff around budgetary and human resources issues at the university. Numerous negotiations with UBC Office of Research Services and University Industry Liaison office and the BC Support Unit ultimately resulted in an agreement, acceptable to all parties, to transfer a portion of funds to Firelight, Inc in keeping with the spirit and intent of the project, and acknowledgement of the roles of Indigenous Research Leads, so that they could recruit and support Indigenous Research Fellows and Community Leads.

We recruited and oriented Indigenous Research Fellows and Project Managers at Firelight and Birth Place Lab. They supported Community Engagement, and the development and distribution of community agreements (Supplemental File S1), project summaries, Community Lead role descriptions (Supplemental Files S2 and S3), consent forms (Supplemental File S4), and data collection methods training documents (see S5) to support community engagement.

Three of the trainees included an Indigenous undergraduate student research assistant, an Indigenous Research Midwife, and a settler medical student research assistant. They collaborated under the Co-PIs supervision to document the research process and procedures, facilitated communications and recorded meetings with communities. They also interviewed the PIs, SC members, and Community Leads over the course of the project to elicit lessons learned around decolonizing birth research. Recognizing the importance of voice-centered relational methods of analyses (28, 29), the quotes presented in this paper are selected to illustrate the findings.

### Settings

This project was envisioned as a companion project to the national Research Examining Stories of Pregnancy and Childbirth in Canada Today (RESPCCT). The Co-PIs on the DBR project were also co-Investigators for RESPCCT. Recognizing timing and funding constraints, we elected to apply a decolonized approach to understanding experiences of respectful care in six Indigenous communities where we had established community relationships. We identified and confirmed five communities across BC: Haida Gwaii, Seabird Island, Port Hardy, and two Nations in the Vancouver urban, and rural Okanagan regions, where Elder Roberta, Rachel Olson, and/or Saraswathi Vedam had existing relationships with midwives, nurses, or community leaders; and Pimicikamak Cree Nation in Manitoba where Wanda Phillips-Beck had established trusting relationships through other research and clinical quality care projects.

A local was designated Community Lead in each participating community. Our team supported them as they led the work to define local research priorities, select culturally appropriate data collection methods (e.g., storytelling, body mapping, birth journals, talking circles), and lead community engagement. Ethical protocols were adapted in consultation with local Elders, Knowledge Keepers and community members to ensure alignment with local governance structures and cultural protocols. Methodologies and findings on respectful care in these and other Indigenous communities have been or will be reported elsewhere (3, 27, 30, 31). Each Nation signed a community agreement that set out the terms of participation in the research project.

The setting of community-based research logically identified the communities in which the researchers set about collecting information about Indigenous families’ experiences in perinatal care. However, for the purposes of this paper, the settings of the research shift to the academic environment, and the reality of attempting a decolonized approach to research that is funded through an academic institution. In this way, the setting moves beyond the “community” and into the realm of meeting room, both real and virtual, with university legal teams, ethics boards, and human resources at the university level. It is noted that the shift in settings ultimately also shifts the power balance or perceptions of the leadership of Indigenous researchers working outside of the academic structures on this project.

## Methods

For the DBR, Indigenous Community Leads within each of those communities conveyed their unique priorities for specific domains of respectful care, modes of data collection, and identified key informants and knowledge keepers in each community. The community leads were instrumental in getting approvals from appropriate governing bodies for us to proceed. The DBR team had a group meeting with all of the community leads to review project goals as a team, discuss the wise ways to collect information in each community on respect, disrespect, and mistreatment in the existing health system during childbearing care. This was also an opportunity to share ideas and successful past ways that the community leads have seen such work be conducted, as well as explore some new opportunities for the work (body mapping, audio/video storytelling, online survey).

The work of the community leads was severely impacted by the pandemic and devastating fires, followed by the discovery of unmarked graves of Indigenous children at the site of a residential school in the participating Okanagan community. One of the communities had a serious COVID outbreak and our Community Lead was pulled into supportive clinical services. The priority for the community and for our team was the health and wellbeing of the community and the DBR team followed the direction of our community leads as to when their communities had the capacity to re-engage with our team.

Nonetheless, highlighting the importance of this topic to Indigenous families, over time all six communities selected data collection methods and five designed community engagement plans. Together the DBR team drafted and revised ethics applications per the identified community preferences. After crossing barriers presented by colonized research processes (detailed in findings), all six community-specific applications were submitted for Elder and Council approvals. Four communities completed data collection: Haida Gwaii (extracted from written birth journals); Seabird Island, (adapted measures from RMC registry to interview Snuneymuxw and Cowichan Nations prompts); Vancouver (community introduced to project and worked with Indigenous storyteller), and Cross Lake held a gathering circle, focus groups and supported online RESPCCT survey completion; and two, Kamloops and Port Hardy, identified preferred methods for future data collection (likely focus groups/ body mapping). (See Table 1 for more information)

**Table 1:** Community-based methods development and data collection.

Research site	Method chosen	Data sharing
Haida Gwaii	Thematic analysis of existing birth journals	High level summary of activities shared with DBR team
Seabird island	Adapted person-centred measures from RESPCCT for internal interviews	High level summary of measures shared with DBR team
Vancouver	Community meeting and work with Indigenous storyteller	High level summary of activities shared with DBR team
Kamloops	Initial exploration of research methods, including body mapping	No data sharing with DBR team
Port hardy	Initial exploration of research methods, including focus groups	No data sharing with DBR team

In addition to the community-based research, qualitative interviews were conducted with project leadership and the research team. These interviews were transcribed and thematically coded. The following sections include anonymized quotes from interviews conducted with Community Leads and project team members during the evaluation phase of the study. Quotes are presented without attribution to specific individuals or communities to protect confidentiality.

## Findings: The need for decolonizing perinatal research

This article recounts the DBR research team’s experiences in launching and implementing an Indigenous-led research project within an academic grant-funded research structure, highlighting both novel community-responsive approaches, how existing institutional systems, processes, and attitudes—shaped by colonial logics—created barriers to conducting decolonizing research. The project facilitated the co-development of validated, culturally relevant measures and data collection approaches that are resonant with both communities and perinatal health researchers. As a result, the DBR team were able to co-develop a roadmap and strategy for Indigenous and other historically marginalized communities to lead or meaningfully participate in maternity care research that promotes autonomy cultural safety, and decolonizes birth research (see Figure 1: Co-Creating a Roadmap to Advance Equity and Inclusion in Perinatal Research and Supplementary File 6: Description of Figure 1).

**Figure 1:**
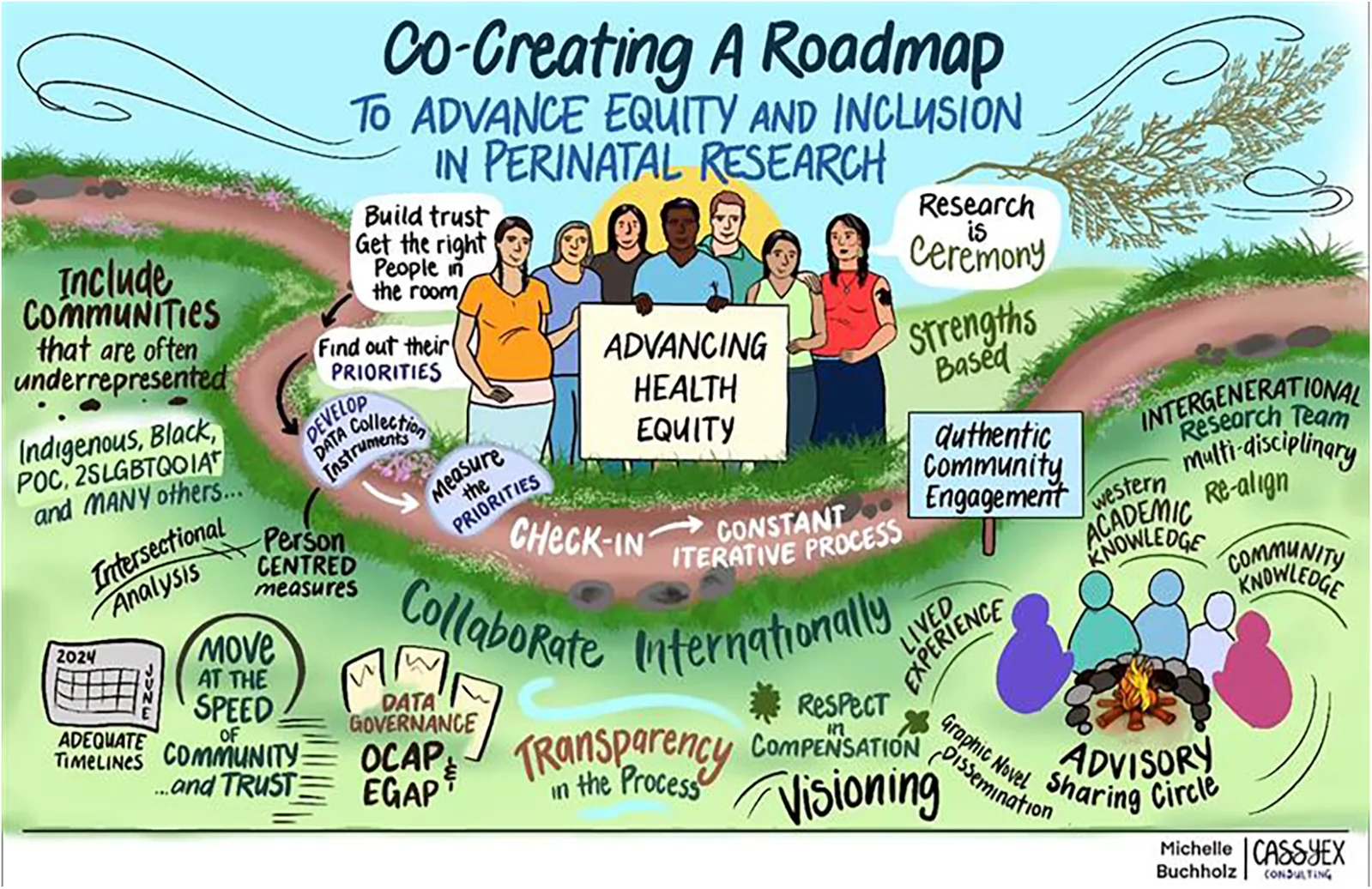
Co-creating a roadmap to advance equity and inclusion in perinatal research. An illustrated conceptual roadmap highlighting key principles for equitable perinatal research.

From a methodological perspective, this project advances patient-oriented research by demonstrating the efficacy and necessity of Indigenous leadership, relational accountability, and flexible, trauma-informed engagement strategies. The approach challenged conventional research paradigms by positioning community members not merely as participants but as co-investigators and decision-makers. The process generated practical outputs including research training materials, adaptable templates for ethics and project governance, and community-specific knowledge mobilization tools such as policy briefs, storytelling dramatizations, and health professional training modules.

### Centering Indigenous research

Indigenous research is grounded in Indigenous worldviews and knowledge systems. Relationality is at the core of Indigenous research including relationships with participants (not “subjects”), communities, the natural world, ancestors, and spirit (32). This deep commitment to relationships engenders deep accountability. It also engenders non-hierarchical approaches where both researchers and research participants all bring specific gifts and knowledge. Researchers are not “experts” but rather act as helpers within a community-centered and participatory approach. Based on this understanding, it is vital that each community plays a central role in the research from conception through implementation and reporting. Indigenous research recognizes that oral and experiential knowledge is rich and valid, and the use of Indigenous languages can strengthen the integrity of research (25). Indigenous research respects community, cultural, and research/data governance protocols (32). Indigenous research is not just research that is done by Indigenous people, nor is it Western research made to look Indigenous. Rather, Indigenous research represents a profoundly different endeavor. In describing the superficial way in which many institutions treat Indigenous research, an Indigenous DBR team member noted, “*It’s just like, ‘we'll call something a sharing circle instead of a focus group and now we've done it*’” (DBR team member, 2024).

The DBR project sought to understand how Indigenous people and families in BC experience birthing through the co-design and application of person-centered, community-responsive measures of respect, disrespect, racism and/or mistreatment over the course of perinatal care (ie. from pre-conception through pregnancy, labour, birth and early parenting). To advance an Indigenous research approach, the DBR team began with two Indigenous researchers collaborating with a non-Indigenous midwife researcher with an academic appointment to co-develop a funding proposal focused on Indigenous-led health services research. We made an explicit commitment to upholding four fundamental values, called the “Four Rs”, namely: respect, relevance, reciprocity, and responsibility (33). These values were the guiding forces behind all decisions related to project design from governance to communications, methods, consent, data analysis, staffing, reporting, and timeframes, among others. However, DBR was situated within the context of an academic grant within the university setting. The shift of setting, as described below, addressed some of the colonial issues that the research team faced throughout the process. It is this processual shift that defined this project and contributes to the broader understanding of what it actually means to decolonize research in Canada.

### Centering Indigenous research leadership and collaboration

Despite significant challenges posed by the COVID-19 pandemic and several community crises (e.g., natural disasters, uncovering of unmarked graves at the sites of former Indian Residential Schools), the research team maintained engagement through virtual tools, flexible timelines, and strong relational commitments. Pivotal to this understanding were the recruited Community Leads, some of them who were midwives, birth workers, and parents themselves. *“Figuring out local champions or leads is an essential process. I think we've done a good job in building those relationships and finding the right people in the communities. I think that we identified some community leaders and people who are really interested in research within the communities, and I think that’s been strength, while it’s taking some time.”* Flexibility was prioritized for the Community Leads themselves, recognizing this project is one position they hold amongst many responsibilities within community; “*acknowledging the realities of their intersectional lives and their lives as having community and family obligations. We build in things like not just food for the participants but for their extended family, or childcare, or time off, or travel money.”.*

“*We've been flexible about how they organize their work within their own communities, trying to let them lead it. Grants often ask you to put in specific amounts of funds in specific lines for the types of people involved. That’s not always realistic, for example in one community there are two Community Leads because they want to share the workload and we were fine with them doing that and sharing the funds. Another community is going to do the data collection in a way that we didn't anticipate and doesn't fit neatly into the focus groups or interviews that we put into the budget line items. So, we've instead given them the package amount for all of the lines and let them decide how to allocate the funds.”.*

One of the Indigenous DBR Co-PI’s describes their experience: “*I always get to this point in my relationships with non-Indigenous researchers where there’s this tension, like ‘we need to get this done, we need to get this done’ and me being like ‘it’s cool, just chill, this is going to happen.’ So, it’s about working on those relationships between researchers and knowing when to let the Indigenous researcher take the lead, and that it’s okay. There’s a point where that control has to shift and you have to trust, even though it doesn't look like things are moving, things are actually moving.”.*

The interdisciplinary research team in the DBR project fostered a deep sense of trust between the research team and the Community Leads, community members, and leadership. Several Community Leads shared explicitly that, though they remain cautious about engaging in research with the University, they were comforted by the central role of an Indigenous organization in the project. Specifically, community partners felt assured that the project would adhere to ethical research principles. For example, in alignment with OCAP® principles—Ownership, Control, Access, and Possession—each participating community retained the authority to determine how their data would be managed. Communities were given the option to: (1) conduct data collection only, (2) collect and retain the raw data with support from Indigenous researchers or BPL team members during analysis, or (3) collect and analyze the data entirely within the community. In each scenario, Firelight, Dr. Phillips-Beck, and the BPL served as collaborators rather than directing the research, offering technical and analytical support only when invited and under community direction. When asked by a community, Firelight or BPL team members performed the analysis in close collaboration with community partners, ensuring that interpretive frameworks reflected community values and priorities. Throughout the project, it became clear that community partners recognized Firelight as a trusted ally with a demonstrated track record of working in respectful, accountable ways with Indigenous peoples, which reinforced confidence that their priorities, protocols, and worldviews would be respected throughout the research process.

### Confronting colonial violence within academic systems

The project team had to navigate several significant external challenges throughout the lifetime of the project. However, from a research team perspective, perhaps the most intractable challenge to the DBR project was the deeply entrenched colonial logics, or structural violence, that emerged in our relationships with academia at every stage. From funding to ethics processes, to institutional oversight and administrative expectations, the research team encountered several challenges that reflect the institution’s continued prioritization of Eurocentric ways of knowing, being, and doing. Despite the project’s focus on decolonizing birth research, these colonial logics persisted in ways that forced the team to navigate conflicting values and priorities to protect our relational, respectful, and holistic research approach. Through these reflections, this article aims to illuminate how colonial violence persists within academic systems and to provide guidance for researchers and institutions seeking to disrupt these structures and reimagine research as a decolonial, relational, and community-centered practice—both within and beyond the academy.

As with many institutions across Canada in the post Truth and Reconciliation (TRC) and post United Nations Declaration on the Rights of Indigenous Peoples (UNDRIP) world, UBC has made many notable commitments to Indigenous peoples. For example, in 2020 the UBC launched an inspired Indigenous Strategic Plan through the Office of Indigenous Strategic Initiatives. The Plan includes a goal to advance Indigenous research by “support[ing] research initiatives that are reciprocal, community-led, legitimize Indigenous ways of knowing and promote Indigenous Peoples’ self-determination” (35). Specifically, Action 13 calls for “Co-develop[ing] research protocols and community-specific ethical research guidelines with interested community partners to ensure students and Faculties are approaching research opportunities with communities in a respectful and formalized manner. This includes the imperative of free, prior and informed consent and protocols on the ownership, control, access and possession of Indigenous data” (31).

Though these are important commitments to be sure, our experience is that implementation at the structural level is lagging; and, critically, the ethics and funding processes at UBC remain deeply colonial, generated and maintained through epistemic violence. Epistemic violence, “refers to the different ways in which violence is exercised in relation to the production, circulation and recognition of knowledge: the denial of epistemic agency for certain subjects, the unacknowledged exploitation of their epistemic resources, their objectification, among many others” (36). Rigid funding and ethics processes at institutions like UBC participate in epistemic violence by prioritizing bureaucratic requirements and institutional control over the needs, values, and priorities of Indigenous researchers and communities. These processes are typically designed to protect and serve the interests of the institution and uphold standardized, Western-centric approaches to research, which can be inherently misaligned with Indigenous ways of knowing, relational accountability, and community-driven research methodologies. They replicate colonial dynamics by placing control over research in the hands of institutions rather than Indigenous peoples and communities. They perpetuate a legacy of extraction and disregard for Indigenous self-determination, positioning Indigenous peoples as subjects of research rather agents of knowledge creation. Decolonizing these processes requires a fundamental shift in power and perspective—one that values relationality, respects Indigenous governance and knowledge systems, and prioritizes community-led approaches at every stage of the research process.

Our DBR research team was relatively successful in shielding community participants from the structural violence that characterized our work with the university. This was accomplished through several means. Firstly, having a strong ally within the institution who was willing to actively challenge the systemic barriers embedded in academic structures was vital to our project’s success. One of the Indigenous Co-Principal Investigators (Co-PIs) highlighted the significant role played by the Birth Place Lab, led by Dr. Saraswathi Vedam, in working to ensure the research was conducted in a way that aligned with decolonizing principles. Reflecting on the lab’s efforts, one Co-PI observed, “*I think Birth Place Lab is pretty hardcore in their approach to trying to take down those institutional barriers for the research. I have so much respect for the fights that they're willing to have about doing research in the right way*” (DBR research team member, 2021). This pushing back was only possible because of Dr. Vedam’s well-established academic record and experience as a seasoned, racialized midwife navigating systems that represent medical hegemony. It is far less likely that a newer and less established academic partner would be familiar with and willing to take the same level of risk in pushing back against colonial policies and processes.

These fights were not merely symbolic; they had tangible impacts on how the research unfolded. The BPL’s willingness to advocate for changes within UBC’s rigid structures became a pivotal moment in building trust with community collaborators. A DBR team member shared how the lab’s efforts to ensure that funds were distributed quickly, equitably, and fairly to community researchers, through Firelight, were instrumental in addressing skepticism from community members. “*[Community] people are skeptical of researchers, especially academic researchers, and they're like, who the hell is this person? Why does she think she can come in and talk about decolonizing birth research? And then I say, well, you know that she fought UBC … that’s what became kind of powerful and important”* (DBR team member, 2024).

### Ethics and research funding

Research ethics are a fundamental part of research processes that are designed to ensure participant autonomy, do no harm, and maximize the benefits (34). The DBR team also recognize that the process and structure of ethics boards are a part of the larger institution constructs that both support and challenge Indigenous-centred research. Funding policies created major obstacles in implementing our project. For example, UBC releasing the project’s funding was contingent on being approved through the university’s ethics process. However, the ethics approval process required signed research agreements from five communities. For communities interested in participating, this requires significant time and effort in advance of any funding being confirmed. It is unreasonable to ask those who have real and deep mistrust of institutions such as universities to “just trust us”. It was always the position of our team that a process built on respect and reciprocity cannot ask community partners to undertake this work without compensation. One DBR team member reflected, “*UBC seemed to understand that we couldn't get [community] permissions until we started to form relationships, and we couldn't start to form relationships without acknowledging the need to pay. We had to figure out how much they would release. But then they held on to the funds …. which made it difficult for me to assign that work to my staff. We were doing a lot of those things without payment*” (DBR research team member, 2021).

Indigenous Co-PI, Dr Phillips-Beck, was able to help navigate the university ethics requirements. A core process to decolonizing this research was engaging all community-based ethics and consenting processes from the outset, which did not mean using a one-size fits all standard consent form, “*There’s a very formulaic UBC standardized template consent form and they were like ‘it has to fit the UBC template*’. Our argument was that the communities the team was working with may have their own consenting process, they may want a different consenting process. Dr. Wanda Phillips-Beck provided templates for consent forms that had been acceptable to Indigenous governments and organizations in previous research that she had conducted that the DBR team then adapted to this project and sent to the BC communities, and they adapted it again for their own community. Yet those forms required “*….a little bit of retrofitting or trying to guess what [UBC] ethics wanted to make it sound good, when this really wasn't the way it would have been done within the Indigenous communities*.” Dr. Phillips-Beck also had established trusting relationships with the Community Lead in the Cross Lake Community and she facilitated introductions, recruitment, and innovative resource allocation when connectivity in that remote region prohibited reliable access to participation via an online survey. Her deep knowledge of respectful and feasible ways to conduct research in that community allowed us to adapt the mode of data collection via the Community Lead carrying an iPad to each home within their own “bubble” in a time of lock-down.

After much negotiation the project team was permitted to move forward with only two of the proposed six communities having signed research agreements. In seeking the two communities, Indigenous research team members leveraged their own personal and professional relationships in the hopes that the promised funding would come through. Even reaching this compromise was a fraught process including one deeply unsettling meeting when the chair of the ethics board took time to explain ethical Indigenous research to one of the Indigenous Co-PIs who has a PhD and 20 years of community-based research knowledge and experience.

The university ethics process does not serve Indigenous-led research teams doing the work in alignment with Indigenous values and principles of decolonized research. While the ethics board included members with Indigenous research expertise, it seemed that the ethics board did not appropriately recognize the community relationships and deep expertise of the DBR team, including researchers working primarily outside of the university system, and the expertise of clinician researchers. In addition, there was not a willingness from those working within the ethics system to support the research team in navigating the process. One DBR member expressed, “*It’s getting just more and more evident to me that the University should have people within the ethics board and the ethics administration process (and they may say that they do) who are really highly trained in working with Indigenous communities, working with minority communities, and who when you come up against these things, they can actually help you navigate and provide solutions. Whereas I think it’s the opposite way around - it always comes back to the individual researchers and the labs to be like ‘OK how do we navigate this’. I think there’s a disconnect there*” (DBR research team member, 2021).

### Allocation and management of research funds

Once the funding was received, the UBC Birth Place Lab was required to manage the funds in line with university policies and procedures, including human resources. The team identified a profoundly qualified candidate with experience in Indigenous birth work, a relatively rare skillset, to act as the project coordinator. However, according to UBC policies, without a master’s degree, the team was unable to pay the candidate appropriately. The DBR team notes that UBC’s own 2020 Strategic Plan committed to advancing research that “legitimiz[es] Indigenous ways of knowing” (35). This excludes, it seems, Indigenous expertise garnered through experiential community and cultural knowledge.

An additional barrier was the lack of urgency to pay community participants, a fact that presented real risks to the relationships between the research team and communities which were fundamental to ethical Indigenous research practices, and ultimately fundamental to the project’s success. One team member shared, “*A big colonizing roadblock is getting money to people in the community. That’s been an issue for me for 20 years of doing research. It never works for people— with honorariums you wait three months to get to get paid*” (DBR research team member, 2021). Many team members felt that the university’s payment processes reflect a paternalism very familiar to Indigenous people in Canada, where Indigenous participants cannot be trusted to fulfill their obligations on their own and must undertake a high administrative burden to “prove” their contributions. Based on the funding process alone, it is simply not possible to claim to support Indigenous research when Indigenous expertise is not recognized and appropriately compensated, when onerous paperwork is required of community people including Elders, and when Indigenous participants are left without pay for long periods of time. Indigenous research requires real action based in respect and reciprocity, including dismantling these common and long-standing institutional barriers.

To avoid payment delays that detract from the relationship building process, the DBR team planned to distribute honoraria and payment to community-based researchers and participants via Firelight, a research consultancy owned in part by our Co-PI Dr. Rachel Olson. Firelight was not bound by institutional restrictions, allowing the team to hire and compensate Indigenous staff based on community-recognized expertise and knowledge, and deliver payments to community participants in a timely fashion. This proposed approach required significant negotiation with UBC’s Office of Research Services and University Industry Liaison Office (UILO). It was UBC’s position that the BPL should hold the funds and subcontract to the two Indigenous PIs. One team member shared, “*We held firm on that fact that we didn’t feel that that was appropriate as Rachel is a co-lead on this project. It was a lot of back and forthing with the lawyers at UILO [University Industry Liaison Office]. We did eventually manage to get an advance on those funds and get them sent across, and that was a big win for the project*” (DBR research team member, 2021). Another shared, “*I think that practically [it] was a good thing for this project, but also it was a very symbolic process that we were going through, in that the University Birth Place Lab received this funding and transferred the majority of that funding to an Indigenous organization. It removed those institutional barriers, and it was the symbolic nature of actually just giving all the money, putting it in the hands of an Indigenous organization that was a very big deal*” (DBR team member, 2021).

To our research team, the level of push back from the university over what amounted to $90,000 was wildly disproportionate. The DBP leadership team suspects that UBC’s discomfort with this proposed innovation was rooted in several facts: firstly, the institution would have to give up a degree of control, and trust that the designated Indigenous Co-PIs would manage funds in ways that ensured achievement of deliverables. Secondly, this model pushes back against the idea that ethical and high-quality research only takes place within academia. The university was much more comfortable serving as a gatekeeper. As one DBR team member shared, “*all this talk about supporting community-based research is only if universities really get to be the ones controlling it*” (2024).

The use of Firelight as a partner in the DBR project represents an important innovation that offers valuable lessons for future work. This partnership demonstrated how leveraging Indigenous-led organizations can create practical solutions to structural challenges while aligning with the principles of decolonizing research. For example, Firelight’s ability to facilitate rapid financial transactions was a particularly critical contribution to the project’s success. A DBR team member shared, “*Once a community coordinator submitted an invoice, we had a very quick turnaround time with a direct deposit system where they would get reimbursed like within 24 h, within a couple of days, they, we just sent the money right to them. So that process became really, really smooth… I think those were all the structural challenges that I felt were probably the most important*” (2024). This efficiency not only met the logistical needs of the project but also demonstrated respect for the time and labour of community partners. Moreover, this partnership highlighted the vital need for universities and funding bodies to recognize that high quality, ethical Indigenous-led research is happening outside of university institutions.

Academic institutions often position themselves as the primary or exclusive site of legitimate research, which is reified through funding frameworks, such as with CIHR, that require funded projects to be led by researchers with academic appointments. If the goal is to support research projects that emerge from Indigenous communities themselves, why is it that communities cannot take on this work? This structure reinforces colonial hierarchies of knowledge, where “expertise” is narrowly defined through Western academic credentials rather than the lived experience, cultural knowledge, and community-based approaches that are central to Indigenous research methodologies. Such requirements exclude many Indigenous people, communities, and organizations who hold deep expertise but may not fit within institutional frameworks. How can a funding agency or university claim to support Indigenous self-determination while mandating an ongoing institutional gatekeeping role that fails to acknowledge Indigenous self determination?.

## Conclusion

The DBR project reveals both the possibilities and the limitations of conducting decolonizing research within academic institutions. Despite public commitments to reconciliation and support for Indigenous research, university systems remained deeply embedded in colonial logics, through restrictive ethics protocols, inflexible funding mechanisms, and entrenched hierarchies of knowledge that often excluded or devalued Indigenous expertise and governance (26). These barriers were not peripheral; they were central challenges that the research team had to continuously navigate.

Partnering with an Indigenous organization, Firelight, and creating a governance structure with two seasoned Indigenous researchers as Co-PIs, proved essential not only for advancing the research but also for protecting community relationships and ensuring alignment with Indigenous values, timelines, and protocols. This collaboration demonstrated that meaningful, ethical, and methodologically rigorous research does not depend on academic affiliation. On the contrary, Indigenous communities and organizations are leading some of the most innovative and accountable research practices, often more effectively than institutions bound by colonial structures.

Firelight’s role in the DBR project demonstrated the value of Indigenous organizations in doing research differently. With Firelight’s involvement, the DBR project demonstrated that meaningful, community-driven research is often better facilitated by organizations that are deeply embedded in and accountable to Indigenous communities. Universities and funding institutions must acknowledge and respect the expertise and ethical leadership of these organizations, recognizing that they often have greater capacity to navigate community relationships, adapt to local contexts, and implement research practices that reflect Indigenous values. In doing so, universities can support a shift towards research that redistributes power and resources and centers Indigenous voices, worldviews, expertise, and leadership.

The reciprocity of the work must be considered throughout to ensure that the team is “*doing research that is relevant for communities and creates information that can actually be put into action*.” Not only the immediate outputs, but from a macro perspective; “*I come from a philosophy that what we do today will impact seven generations in the future. My hope for the Decolonizing Birth Research project is that in seven generations we will have returned some measure of birthing to our communities. That we are in control of our childbirth practices, that we are telling our own stories, that we are practicing our traditions and our ceremonies that really grounded us in who we are as the first people of this land. Those values that we carry forward in this research I hope will be threaded throughout the generations and will make a huge difference in the future. We may not see it in our lifetime, we may see slight changes or small changes, but my hope is that in seven generations it will be completely different*.” (Indigenous Co-PI).

This project challenges the assumption that the academy is the only— or even the most appropriate— site for ethical Indigenous research. It calls on academic institutions to not only reflect on their colonial foundations but to take concrete action: redistributing power and resources, removing structural barriers, recognizing birth and bodily sovereignty, and supporting Indigenous leadership of health equity research without co-optation or control. Decolonizing research means reimagining research itself—not as an extractive academic exercise, but as a relational, community-centered practice rooted in respect, reciprocity, and Indigenous self-determination.

## Data Availability

The original contributions presented in the study are included in the article/Supplementary Material, further inquiries can be directed to the corresponding author.
