# Supplementary material for: Start with the truth: lessons from the decolonizing birth research project
Source: Front Glob Womens Health. 2026 Oct 30;7:1780730. doi: 10.3389/fgwh.2026.1780730 (PMC13759479; doi:10.3389/fgwh.2026.1780730)
Supplement: Supplementary file 1 [file Datasheet1.pdf]

**Statement of Participation by ____________________ FIRST NATION Nation Inc.
and Decolonizing Birth Research Team,
Re: Decolonizing Birth Research Project**

________________ **FIRST NATION** agrees to participate in the research project entitled, "*Decolonizing Birth Research–Indigenous Researchers, Clinicians, and Community Experts measuring respect, disrespect and mistreatment in Indigenous childbearing communities*", funded by a grant from the Measurement Cluster at the BC Support Unit from **December 1st 2019 – December 31st 2021**.

The **Decolonizing Birth Research Team** and _______________ **FIRST NATION** will be responsible for the following items to ensure the project assists the First Nation to its full potential. This Statement of Participation is a document of working relations, and not a legal document. In future, legally binding documents might be developed, e.g. a data sharing agreement that outlines how principles of OCAP will be met. Such agreements will be drafted according to templates developed by Alberta First Nations Information Governance Centre or equivalent templates and reflect community preferences and interpretations of OCAP.

The **Research Team (comprised of members of The Firelight Group, the Birth Place Lab at the University of British Columbia, and Wanda Phillips-Beck an Indigenous Research Scholar affiliated with the *First Nations Health and Social Secretariat of* Manitoba*)*** agree to the following**:**

1. All team members will complete the 7-hour Principles of OCAP course
2. All team members will participate in group and individual learning activities around anti-Indigenous racism
3. We will compensate participants fairly; honoraria are available for Elders, community members and community Leads who agree to participate in project activities.
4. Coordinate the overall implementation of the research project, under the guidance of Community Leads and with permissions from the Elders in the First Nation.
5. Conduct virtual visits (via phone, skype or zoom) on a monthly basis or agreed upon scheduled occasions and keep open communication with local research assistants, Community Leads, Health Director (HD) and/or Elders and Knowledge Keepers as designated by the community.
6. Actively support the community's participation in the project via phone, zoom, email, mail, and other forms of remote communication for the duration of the project
7. Provide information to the HD and/or Elders in the community via email or newsletters regarding the status of the research project

8. University of British Columbia will reimburse the community for all expenses for travel and accommodations for the Health Director, Community Leads, Elders, or community representatives in the performance of duties directly related to research project as per University of British Columbia financial policies and travel rates.
9. Seek review and approval of draft publications or reports by community leadership.
10. Co-develop with the community a road map for best practices for researchers to ensure that Indigenous ways of knowing drive Indigenous ways of doing, especially when conducting health research.
11. Collaborate as partners with the community engagement phases, to enact culturally safe ways of conducting the research, and develop procedures that ensure that ownership of the data remains within the community.
12. Recognize that the participating First Nation has intellectual property rights over publications, reports, acknowledgements of their data and the requirement to present results/research to First Nation's first, pre-publication notices with the right of dissent, and correct attribution of First Nation data in publications and research.
13. That the First Nations Data shall be used and disclosed only and strictly for the purposes specified in this Agreement.

The ______________________ **FIRST NATION** agrees to:

1. Provide oversight and ensure that the community-based project as outlined in **Project Summary** is on schedule.
2. Arrange for the HD, Community Leads, and/or Elders to attend workshops/meetings directly related to research project.
3. Collaborate as partners with the research team to provide leadership throughout the community engagement and data stewardship phases.
4. Support the project locally encouraging community members, staff, Elders, youth, and leadership to actively contribute to and participate in the research project.
5. Contribute to the plans for analysis of data on respect and disrespect during childbearing care from the community.
6. Co-develop with the Research Team a road map for best practices for researchers to ensure that Indigenous ways of knowing drive Indigenous ways of doing, especially when conducting health research.
7. Review findings and share their insights with the research team at the Firelight Group, University of British Columbia and research team member with the First Nations Health and Social Secretariat of Manitoba.

8. Share with the Research Team community preferences for key messages, audiences, and modes of knowledge translation for the results for this study.
9. Co-develop any publications or reports, and facilitate the review and approval of draft publications or reports by community leadership.
10. Contribute to dissemination of the results of the study via the community's established local and provincial networks.

The Parties recognize that this Agreement constitutes a living document, with the intention that necessary amendments be made according to the terms herein, as circumstances warrant.

We have read and agree to the above:

____

UBC/Firelight/FNHSSM signatory **Date** **Witness**

____

[NAME/title/Authorized signatory/FN] **Date** **Witness**
