# Supplementary material for: Start with the truth: lessons from the decolonizing birth research project
Source: Front Glob Womens Health. 2026 Oct 30;7:1780730. doi: 10.3389/fgwh.2026.1780730 (PMC13759479; doi:10.3389/fgwh.2026.1780730)
Supplement: Supplementary file 2 [file Datasheet2.pdf]

Summary of project

The overarching goal for this study is to improve the childbirth experiences of Indigenous people who live in remote or rural areas, young parents, and those who experience multiple life challenges (such as incarceration, relocation, substance use, housing instability and/or poverty). Funded by the BC Support Unit for People and Patient-Oriented Research, this project aims to to develop person-centered measures of respect, disrespect, racism and/or mistreatment as experienced by Indigenous families during pregnancy and childbirth.

This project is led by Indigenous researchers, Dr. Rachel Olson, Co-Director of the Firelight Group and Hon Wanda Phillips-Beck, Seven Generations Scholar, First Nations Health and Social Secretariat of Manitoba, and with the support of Elder Roberta Price and Dr. Saraswathi Vedam, Principal of the Birth Place Lab. With their collective expertise guiding the participatory methods for this research, we invite community members to engage in every step of the research process from visioning how to collect data, leading data collection in community, helping the team to interpret the results and taking the findings back to the communities. We recognize the need to ask for input in culturally safe ways and multiple ways of collecting information such as online survey, talking circles, voice or video recordings made by women who want to share a story through a mobile app, and/or one on one conversations with trusted community leaders and Elders. We will seek guidance and leadership from Indigenous Knowledge Keepers and Elders on respectful ways to collect information in their community members.

We acknowledge that Indigenous Knowledge Keepers and Elders are integral to our project and must be involved and provide leadership throughout the community engagement phases, to ensure that Indigenous ways of knowing drive Indigenous ways of doing. They also play an important role in providing safe spaces for community members and opportunities for healing during difficult times. This is critical when addressing issues that are so closely linked to colonial histories, harms, and the impacts of systemic discrimination. To that end, we have developed this proposal to demonstrate wiser practices to decolonize health research within the broader context of efforts towards Indigenous self-determination.

In summary, we will co-develop a roadmap and strategy for Indigenous communities to lead or meaningfully participate in maternity care research that promotes autonomy cultural safety, and decolonizes birth research. Our team is committed to community-led knowledge translation of our findings, choosing if, what, and how to tell these stories to health policy leaders about health services, healthcare accessibility, and health professional education towards improved equity and quality of care for all families.

To find out more about the Firelight Group, please go to
https://firelight.ca/

To find out more about the work of the Birth Place Lab, please go to
https://www.birthplacelab.org/
