# Supplementary material for: Start with the truth: lessons from the decolonizing birth research project
Source: Front Glob Womens Health. 2026 Oct 30;7:1780730. doi: 10.3389/fgwh.2026.1780730 (PMC13759479; doi:10.3389/fgwh.2026.1780730)
Supplement: Supplementary file 3 [file Datasheet3.pdf]

Job description for Community Lead

This project aims to develop person-centered measures of respect, disrespect, racism and/or mistreatment as experienced by Indigenous families during pregnancy and childbirth. Together with 5 Indigenous communities and/or Nations in BC, and one in Manitoba, we will co-develop a roadmap and strategy for Indigenous people to lead or meaningfully participate in research that promotes autonomy, cultural safety, and respect in perinatal services.

The Community Lead will invite community members to engage in every step of the research process from visioning how to collect data, leading data collection in community, helping the team to interpret the results and taking the findings back to the communities. Methods for data collection may include participation in an online survey, talking circles, voice or video recordings made by women who want to share a story through a mobile app, body mapping, and/or one on one conversations with trusted community leaders and Elders. The Community Lead will seek guidance and leadership from Indigenous Knowledge Keepers and Elders on respectful ways to collect information in their community members.

This project is led by Indigenous researchers, Dr. Rachel Olson, Co-Director of the Firelight Group and Hon Wanda Phillips-Beck, Seven Generations Scholar, First Nations Health and Social Secretariat of Manitoba, and with the support of Elder Roberta Price and Dr. Saraswathi Vedam, Principal of the Birth Place Lab.

Roles and Responsibilities may include

- Help to **distribute or share information on the Decolonizing Birth Research study** through neighbourhood, community or regional networks
- **Provide in-person support** for local recruitment and data collection events and strategies (phone interviews, ipad recordings, online focus groups, talking circles)
- Coordinate with Study Leads, Indigenous Research Fellow, and Birth Place Lab team to meet with local groups and organizations
- Attend orientation and monthly Community Lead meetings to share best practices, insights, ideas and strategies
- Keep a record of local contacts and communications, including Elders, Knowledge Keepers, and community partners
- Work with the Indigenous Research Fellow to track engagement, identify key knowledge keepers and decision makers in community, secure permissions and support local events/ development of recruitment materials
- Be a RESPCCT Study Champion – distribute link and social media materials, provide information on the study, support local families to access survey, help with story telling
- Support community members in the knowledge translation phase to review findings, key lessons, and teachings
- Document key steps, facilitators and barriers to studying respectful health care in the community.
- Collaborate with Study Team to develop and present findings to the community and external stakeholders through reports, manuscripts, policy papers, and/or community facing and arts-based KT tools

Qualifications

- Have an interest in health equity research, and health services with a focus on Indigenous health and well being
- Willingness to engage with best practice research materials, ethics, and OCAP trainings
- Be familiar how pregnancy care and who provides services in the region
- Be willing to establish relationships with doctors/nurses/midwives and other maternity care service providers, community agencies, families, and leaders at institutions that provide pregnancy and childbearing care services.
- Be able to represent and/or understand the needs, perspectives, and priorities of women and families from your own community
- Be willing to work respectfully with other maternity care stakeholders including health care providers, researchers, and policy makers
- Be interested in improving maternity care in Canada for all who experience childbirth care in all settings (office, clinic, home, community centre, birth centre, or hospital)

To find out more about the Firelight Group, please go to

https://firelight.ca/

To find out more about the work of the Birth Place Lab, please go to
https://www.birthplacelab.org/

Contact us:

engage.birthplacelab@ubc.ca - www.birthplacelab.org – (604) 875-2424 ext. 5771
