# Supplementary material for: Start with the truth: lessons from the decolonizing birth research project
Source: Front Glob Womens Health. 2026 Oct 30;7:1780730. doi: 10.3389/fgwh.2026.1780730 (PMC13759479; doi:10.3389/fgwh.2026.1780730)
Supplement: Supplementary file 4 [file Datasheet4.pdf]

Decolonizing Birth Research
Indigenous researchers, clinicians, and communities
measuring respect during childbearing

Declaration of Informed Consent and Permission to Use Information

I (name) ____on this day (complete date) ______________________, give permission for the researchers from the Firelight Group or the Birth Place Lab at UBC and to interview me for the *Mapping Sovereign Birth project*.

I understand that Birthing Sovereignty hosting this study and that Firelight Group and the Birth Place Lab at UBC have been engaged as the researchers. The goal for this study is to improve the childbearing experiences of Indigenous people. Funded by the WWW Kellogg Foundation, this project seeks **How are Indigenous communities reclaiming birth?**

a. In what ways are Indigenous communities healing their experiences of pregnancy and birth?

b. What assets are necessary for restoring Indigenous birthing ways of knowing and being around pregnancy and childbirth…?

The team will also work with my community to co-develop a roadmap for Indigenous communities to lead or meaningfully participate in maternity care research that promotes autonomy and cultural safety, and decolonizes birth research.

By agreeing to participate,:

(a) I consent to have my words and responses recorded in notes, and using audio recording equipment.

(b) I am free to not respond to questions that may be asked and I am free to end the interview at any time.

(c) I understant that the community will maintain the intellectual property rights over the data and will co-interpret and approve the findings of the research.

(d) I understand that the researchers and community leaders will co-develop the information in reports submitted to the funding agency (SPOR-PCM), and in future publications, or tools that that are used to inform the public, clinicians, health care systems, or policy makers.

(e) .

Please indicate whether or not you would like your name to appear in reports (circle one):

YES **NO**

For more information, please contact one of the Co-Investigators on the project: Rachel Olson, Saraswathi Vedam, Wanda Phillips-Beck

Dr Saraswathi Vedam, Professor, UBC Midwifery. Principal Investigator at the Birth Place Lab.
saraswathi.vedam@ubc.ca. +1 (604) 875-2424 x 5831

Dr Rachel Olson, Ph.D. Social Anthropology, Director, The Firelight Group.
rachel.olson@thefirelightgroup.com. +1 (604) 563-2245

Wanda Philips-Beck , RN, PhDc Clinical Specialist, First Nations Health Manitoba
wphillips-beck@fnhssm.com>
