# Supplementary material for: Start with the truth: lessons from the decolonizing birth research project
Source: Front Glob Womens Health. 2026 Oct 30;7:1780730. doi: 10.3389/fgwh.2026.1780730 (PMC13759479; doi:10.3389/fgwh.2026.1780730)
Supplement: Supplementary file 5 [file Datasheet5.pdf]

Prepared By:	UBC Birth Place Lab and Firelight Research Inc.
Submitted To:	Community Coordinators
Date:	October 29th, 2020
**RE:**	**Decolonizing Birth Research – Virtual Community Engagement Options**

This memorandum provides a summary of the Decolonizing Birth Research project, community coordinator roles and responsibilities, an overview of the research process, timeline, and budget available, as well as suggestive community engagement and data collection tools for use or reference by the community leads.

Background

The Decolonizing Birth Research project aims to develop person-centred measures of respect, disrespect, racism and/or mistreatment as experienced by Indigenous families during pregnancy and childbirth. This project is led by Indigenous researchers, Dr. Rachel Olson, Co-Director of The Firelight Group, Dr. Wanda Philips-Beck, Seven Generations Scholar and Director of the First Nations Health and Social Secretariat of Manitoba, and with support of Elder Roberta Price and Dr. Saraswathi Vedam, Principal of the Birthplace Lab.

This study is a participatory action research project, so there are also six Community Leads actively participating, informing, and leading each stage of the project, from visioning how to collect data, community engagement and data collection in their communities, interpreting the results, and taking the findings back to the communities with the principal investigators. The ethics approvals for this project arefor recruitment within the following regions, however, each Community Lead has the flexibility to engage who they see fit within the regions (i.e. Okanagan – Kamloops or a specific Indigenous community):

- Okanagan, British Columbia
- Haida Gwaii, British Columbia
- Vancouver, British Columbia
- Seabird Island, British Columbia
- Port Hardy, British Columbia
- Cross Lake, Manitoba

More information on the goals and objectives of the Decolonizing Birth Research project are provided in Appendix A alongside the community coordinator job description provided in Appendix B.

Project Overview

This section summarizes the key steps and tasks associated with the Community Lead's roles in this project.

1. *Identify the community and gain approval from leadership*

While we have UBC Ethics approval to engage the previously mentioned geographical areas, each Community Lead will identify which specific community(s) to engage and follow their community's appropriate ethical permission procedures. A community agreement template is provided in Appendix C. If you have not yet submitted a signed Community Agreement, adapting the template to the local context and getting signatures of Elders or decision makers, and sending the completed form back to Firelight and the Birth Place Lab is the first step.

2. *Identify the research domains and questions*

As previously mentioned, the goal of this research project is for Indigenous service users to inform the development of person-centered measures of respect, support, disrespect, racism, and/or mistreatment that can capture the experiences of Indigenous families during pregnancy and childbirth. This project is a sister-study to the national RESPCCT study which is collecting information through an online survey on peoples experience when accessing pregnancy and childbirth care in Canada. The RESPCCT survey asks people to share their experiences across at the following 12 topics:

- Autonomy
- Responsiveness of Healthcare Providers
- Choice of Evidence-Based Care Options
- Cultural Safety and Family Engagement
- Health System Conditions and Constraints (Human Resources)
- Health Systems Conditions and Constraints (Physical)
- Information and Consent
- The Mothers on Respect index (MORi)
- Non-Verbal Communication
- Patient Reactions to Experiences of Care
- Physical Exams and Procedures
- Physical Mistreatment
- Privacy and Confidentiality
- Stigma and Discrimination
- Supportive Behaviours of Healthcare providers
- Verbal Communication
- Verbal Mistreatment

Some example statements that participants agree or disagree to in the above domains include:

- **Cultural Safety and Family Engagement:**
  "The health care team supported my cultural or family traditions"

"I was allowed to practice cultural rituals in the facility"
"I was able to have exactly the people I wanted with me during labor and birth"
"Finding a midwife or doctor who shared my heritage, race, ethnic or cultural background is important to me"

- **Verbal Mistreatment**:
  "Did you feel the doctors, nurses, or other health providers shouted at you, scolded, insulted, threatened, or talked to you rudely?"
  "The health worker or other staff threatened that if you did not comply, you or your baby would have poor outcome"
  "Healthcare provider(s) threatened to give treatment I did not want"
  "The health worker or other staff threatened to withhold care from you or your baby"

For this project, the community leads may use the RESPCCT study domains and adapt or revise the statements as they see as relevant to their community and community priorities. The most important question to ask, as Community Leads, is whether or not the domains and questions are relevant or appropriate to ask within each unique community, and whether it aligns with the community's current experiences and priorities.

3. *Organize community engagement*

How to engage the community and collect data for this project is entirely up to the Community Leads. This section provides a high-level overview of various methods, the pros and cons of each method, and other special considerations. The methods provided are only meant to be examples, to present options and tools for thinking and organizing community engagement. An important question to ask oneself is "what is the best way for me and my community to talk about this topic?".

a) Survey

Questionnaires or surveys can be distributed to community members to assess their needs, allow them to voice their opinions on issues of concern, and obtain feedback. Depending on the purpose, scope, and who is involved, questionnaires can be targeted toward a certain population group (e.g., youth or women) within the community or designed for the entire community. Questionnaires can be web-based, administered over the telephone, in-person, or via mail. Firelight and the Birth Place Lab can help to create a survey if that is you're your community chooses.

Pros

- ✓ Surveys can be administered virtually with little to no adaptations.
- ✓ Surveys can reach a large amount of people.

Cons

- ✗ Surveys sometimes lack context and dialogue and cannot capture the full picture.

Key considerations:

- ✓ Questionnaires should be clear and concise. This will allow for efficient data collation and analysis of community members' responses.
- ✓ Questionnaires can be targeted to the entire community or they can be sent to a specific community group.
- ✓ It is equally important to ensure the number of responses adequately represents the opinions of the group being surveyed.
- ✓ Questionnaires are a good follow-up engagement option in addition to focus groups and community public forums.
- ✓ Some research topics are highly sensitive, therefore, questions should be carefully curated, with available aftercare supports attached.

Examples:

You can see 210 sample validated questions here: www.birthplacelab.org/rmc-registry

Some of those items were used in the RESPCCT survey:

The RESPCCT survey: https://www.respcct.ca/

b) Individual Interviews

One-on-one interviews are conducted in the form of discussions between an interviewer and a single individual (also called the interviewee). Compared to a focus groups, one-on-one interviews allow the interviewer and the interviewee to have more in-depth discussions. For example, an interviewee is more likely to express their opinions about sensitive or personal issues in a one-on-one setting compared to a group setting with other community members. One-on-one interviews can be structured or semi-structured and can be conducted in-person or remotely (e.g., over the phone, Zoom, Google Meet, etc.).

Pros

- ✓ One-on-one interviews can provide detailed information about the topic of interest.
- ✓ At the end of the interviews, all the information from each interview can be combined to create a clear picture of topic.
- ✓ Compared to focus groups, it is less likely that group dynamics will negatively impact interviewees in a one-on-one interview setting.

- ✓ One-on-one interviews can provide a safe space where participants can freely confide in the interviewer. The interview can progress at a pace that is suitable for each participant.

Cons

- ✗ One-on-one interviews can be more time-consuming and less cost-effective when seeking input from a large group of people.
- ✗ In a one-on-one interview setting, the interviewer does not have the benefit of building on the group conversation and flow that comes from a focus group.

c) Focus Groups

Focus groups or talking circles can be structured or semi-structured group discussions with a limited number of individuals. The goal of a focus group is to seek feedback from a people with lived experiences who can shed light on what will be effective and appropriate for their community in general. The group dynamic helps members play off each other's ideas and through discussion focus group, attendees can brainstorm and reflect on key topics. The group is led through a series of questions by a facilitator and the feedback is usually recorded by an audio recorder and/or a notetaker. These interviews can be undertaken both in-person and/or remotely. For virtual focus groups, suggested online platforms for conducting remote meetings include Zoom, Google Meet, and GoToMeeting. Firelight and the Birth Place Lab can help to set these up and help you to understand how to lead the groups if you need that support.

Pros

- ✓ Focus groups offer key benefits of allowing community members to express their views and opinions about an issue, as well as to brainstorm collectively and stimulate new ideas for each other.
- ✓ Promotes rich discussions and insightful understanding of complex issues and situations, which cannot be gathered from standard surveys or large community forums.
- ✓ It is also more affordable to run a meeting with several participants than it is to run multiple one-on-one interviews.

Cons

- ✗ Some participants may not feel comfortable talking in a group setting, especially if the focus group takes place virtually. It is possible to also have follow-up interviews with key participants that perhaps had more information to share than the focus group allowed for.

Key considerations:

- Set the meeting up so members feel comfortable expressing their ideas. Ice breakers, jokes, ceremony, etc. are useful ways to create a comfortable setting.
- In some cases, particular groups may be more vocal (such as elders or men) or hold stronger positions about certain issues than others (e.g., women or youth). For a more inclusive process that represents all interests, some researchers may consider conducting separate focus groups for elders and knowledge holders, women, and youth, or utilizing other engagement tools.
- Consider a mix of engagement options in addition to the focus groups, for the best outcomes. Survey questionnaires, one-on-one interviews, and/or community forums are excellent follow-up options to focus groups.
- Participants may need support in accessing and using online platforms.
- Focus groups can also be creatively adapted to be more comfortable and engaging for participants by incorporating more of a talking circle approach.
- For virtual focus groups, facilitation tools such as “dotmocracy” and “jamboards” can be combined to support and promote wider participation, particularly from less outspoken participants.

d) Indigenous engagement variations and tools

- **Dotmocacry** – various colored sticky notes (e.g. red, yellow, green) may be handed out to participants to right on for where they prioritize something or agree/disagree on issues or topics. Depending on the research project, 3-5 questions may be asked and given a designated color, for which participants can have the opportunity to write their thoughts on the sticky note. In face-face focus groups or meetings, all the sticky notes can be gathered and posted onto a wall. This provides a visually opportunity of what matters most to community members. Virtual adaptions can include using a jamboard that is somewhat like virtual sticky notes: https://chrome.google.com/webstore/detail/jamboard/ihacalceahhliihnhclmjjghadnhhnoc
- **Sewing and beading circle** - women may come together to openly and safely discuss what respectful maternity care means and looks to them.
- **Land-based engagement** – going out on the land, walking or sitting in a cultural important space may promote a safer, more comfortable environment for participants. Note: it may require multiple notetakers to effectively record such engagement.
- **Community gathering** - Hosting a community gathering that seeks to bring members together may increase participation in any of the above engagement methods. Whether the gathering is a family carnival night, movie night, dinner, etc., can be a good opportunity to introduce the research and invite members to

participate. The survey could be provided at the gathering, a separate safe space for them to fill out if they'd like, or to participate in an individual interview.

e) Body mapping

Body mapping is an arts-based tool that focus on the body as a way to represent experiences lived through the body. It can be a great tool for participant to share their stories in a way that they feel most comfortable and safest communicating through visual means. Exercises involved in body mapping includes tracing full size image of a body, drawing where you come from, your hopes for the future, and painting your supports. It can utilize drawing and painting exercises, visualization, group discussions, and individual quiet times for reflection. There are plenty of ways to adapt the process to suite the communities needs and priorities. Commonly, a facilitator may go through various probing questions that participants may reflect upon and draw, and then wrapping up with a group discussion about participants body maps and how they felt about the process.

Pros

- ✓ Body-map storytelling can offer participants a low pressure opportunity to share their stories around sensitive topics.
- ✓ It can also be a powerful or healing exercise for many, sharing stories, insights, and connections in a way some may never have before.

Cons

- ✗ The use of the participants body map's will need to be clearly communicated and agreed upon.
- ✗ The process might be triggering for some participants, so extra attentiveness and sensitivity to participants comfort is essential.

Key considerations:

- ✓ Body maps are intimate and personal. It is important to inform and remind participants that it is up to them how much they want to reveal and remind them what information they may want to protect for their own privacy.
- ✓ Appropriate after-care supports and services should be made available for those if needed.
- ✓ This approach has the potential to be adapted online similarly to focus groups. Supplies (the body drawing and art tools) should be provided to everyone prior to, and additional care should be taken to ensure participants can participate in a safe space. Overall, it is best to be done in-person, in a gymnasium or community centre.

Examples:

Aboriginal Midwifery Toolkit Chapter 6: Understanding impacts through body mapping art workshop: https://indigenousmidwifery.ca/wp-content/uploads/2018/10/Aboriginal-Midwifery-Toolkit.pdf

4. *Reporting*

After community engagement is conducted and data is collected, the research team will collaboratively work with and support the community coordinators in organizing all the information and developing the best approach to communicate the information.

Funding

Each of the six community coordinators will receive a $5,000 salary for their work on this project. There are additional funds set aside to support organizing and facilitating community engagement. Below is an overview of what is available. Note these are not necessarily fixed amounts and that there is some flexibility for allocating these funds.

- Honoraria for community participants
  - $2000 to be distributed as each community sees fit
  - E.g. $200 per person x 10 participants
- Honoraria for Elders
  - $600 to be distributed as each community sees
  - E.g. $300 x 2 Elders
- Flow through
  - $2,000 to be distributed as each community sees
  - E.g. 10 hours of Elder's review and guidance at $200/hr
