# Supplementary material for: Start with the truth: lessons from the decolonizing birth research project
Source: Front Glob Womens Health. 2026 Oct 30;7:1780730. doi: 10.3389/fgwh.2026.1780730 (PMC13759479; doi:10.3389/fgwh.2026.1780730)
Supplement: Supplementary file 6 [file Datasheet6.pdf]

Supplementary File 6: Description of Figure 1, Co-Creating A Roadmap To Advance Equity and Inclusion in Perinatal Research

Figure 1 is a vibrant, illustrated infographic titled “Co-Creating A Roadmap To Advance Equity and Inclusion in Perinatal Research.” The illustration uses warm, earthy colors, such as greens, yellows, oranges, and browns, to create an inviting and community-oriented atmosphere. The overall layout resembles a winding path or journey, with signposts and visual markers guiding the viewer through key concepts and actions.

At the center of the image, a diverse group of people, reflecting different races, genders, and identities, stand together holding a large banner that reads “ADVANCING HEALTH EQUITY.” The figures are drawn in rich, warm tones, and their collective stance conveys unity and shared purpose.

Surrounding this central group are clusters of phrases and small illustrative icons arranged along the winding pathway and within the soft green grass that borders it. These clusters, presented in hand-drawn lettering and warm color accents, each highlight a principle or step in the proposed roadmap.

Left side: This section emphasizes the inclusion of communities that have been historically underrepresented in research, including Indigenous, Black, People of Color, and 2SLGBTQQIA+ communities. The text appears in bright, contrasting colors to draw attention to concepts such as intersectional analysis, person-centered measures, and the importance of moving at the pace of community trust. Adequate timelines, marked as 2024, are also noted.

Middle path: The central pathway illustrates a collaborative research process. It includes steps such as building trust, bringing the right people together, identifying community priorities, developing data collection tools, and measuring what communities identify as meaningful. These elements are depicted in warm yellows and oranges, reinforcing the idea of ongoing movement and iteration. The pathway emphasizes the need for continuous check-ins and adaptation.

Bottom section: This area focuses on collaboration and governance. Terms such as international collaboration, data governance frameworks (OCAP and EGAP), transparency, respectful compensation, lived experience, and the use of graphic note-taking are presented in grounded, earthy tones. These colors help convey stability, accountability, and shared responsibility.

Right side: The right portion of the infographic highlights cultural and structural transformation. Phrases such as “Research is Ceremony,” “Strengths-Based,” “Authentic Community Engagement,” and “Intergenerational Research Team” appear in warm, uplifting colors. This section also emphasizes the integration of Western academic knowledge with community knowledge and includes an advisory sharing circle, depicted in soft, harmonious tones to symbolize relationality and collective wisdom.
